# A social-ecological approach to support equitable land use decision-making

**DOI:** 10.1007/s13280-024-02056-x

**Published:** 2024-08-02

**Authors:** Maria Brück, Felipe Benra, Dula Wakassa Duguma, Joern Fischer, Tolera Senbeto Jiren, Elizabeth A. Law, Manuel Pacheco-Romero, Jannik Schultner, David J. Abson

**Affiliations:** 1 Social-Ecological Systems Institute, Leuphana University Lüneburg, Universitätsallee 1, 21335 Lüneburg, Germany; 2 Working Conservation Consulting, 1st Avenue, Fernie, BC V0B1M0 Canada; 3 Andalusian Center for the Assessment and Monitoring of Global Change (CAESCG), Department of Biology and Geology, University of Almería, Almería, Spain; 4 Earth Systems and Global Change Group, Wageningen University & Research, Droevendaalsesteeg 3, 6708 PB Wageningen, The Netherlands

**Keywords:** Disaggregation, Ecosystem services, Equity, Land use change, Scenario planning, Social-ecological systems

## Abstract

Human-driven land use change can result in unequitable outcomes in the provision and appropriation of ecosystem services (ES). To better address equity-related effects of land use change in decision-making, analyses of land use and ES changes under different land use management alternatives should incorporate ecological and social information and take a disaggregated approach to ES analysis. Because such approaches are still scarce in the literature, we present a generalized social-ecological approach to support equitable land use decision-making (in terms of process and outcomes) and an example of its application to a case study in southwestern Ethiopia. We propose a six-step approach that combines scenario planning with equity-focused, disaggregated analyses of ES. Its application in our study area made equity-related effects of land use change explicit through the recognition of different beneficiary groups, value types, and spatial locations. We recommend the application of our approach in other contexts, especially in the Global South.

**Supplementary Information:**

The online version contains supplementary material available at 10.1007/s13280-024-02056-x.

## Introduction

Human land use is changing in rural areas around the world, and it is not only a key driver of biodiversity loss, but also affects the provision and appropriation of ecosystem services (ES), i.e., the benefits that people obtain from nature (Millennium Ecosystem Assessment 2005; Quintas-Soriano et al. 2016; Díaz et al. 2019). ES research is still heavily focused on assessing aggregated ES provision or aggregated well-being in relation to possible land use options (Rosa et al. 2017; Mandle et al. 2020), but changes and trade-offs in ES provision and appropriation can create winners and losers (Rodriguez et al. 2006; Carpenter et al. 2009; Cord et al. 2017).

This relates to the concept of equity, which we understand as a multidimensional concept of ethical implications and social justice that is principally concerned with relationships between people, and with their relative circumstances (McDermott et al. 2013; Loos et al. 2022). Equity is commonly analyzed along three dimensions, namely that of distributional equity, i.e., how resources, costs, and benefits are allocated or shared among people and groups, recognitional equity, i.e., who holds which different values, identities, rights and preferences, and procedural equity, i.e., who is involved, and how, in decision-making and political processes (Schlosberg 2007; Fraser 2009; Leach et al. 2018). Unequitable outcomes in ES provision and appropriation, especially in the case of provisioning ES, can result from, among others, power dynamics, value trade-offs, or spatial dynamics, with implications for distributional, recognitional, and procedural equity (Schlosberg 2007; Fraser 2009; Langemeyer and Connolly 2020), as well as for human well-being and sustainable resource use (Leach et al. 2018; Loos et al. 2022). For example, power dynamics, demographic and socioeconomic factors or geographical location, as well as values and priorities can influence people’s access to provisioning ES and the benefits associated with this (Dorresteijn et al. 2017; Schultner et al. 2021).

Scenario planning and analysis of ES provision and appropriation can be useful to anticipate equity-related effects of land use change, and ES are thus gaining importance as key response variables in scenario analysis (Plieninger et al. 2013; Arkema et al. 2015; Felipe-Lucia et al. 2022). According to the literature, ES research in general, and (ES-based) scenario planning and analysis, could improve in multiple ways, in order to better assess and address equity issues, and to be more relevant to decision-makers. For example, the literature prominently discusses the following four suggestions: the integration of ecological and social information (Fischer et al. 2017; Mandle et al. 2020; Felipe-Lucia et al. 2022); the inclusion of disaggregated analyses of beneficiaries and power dynamics (Oteros-Rozas et al. 2015; Berbés-Blázquez et al. 2016; Rieb et al. 2017); the use of multimetric valuation (Rieb et al. 2017; Chan and Satterfield 2020); and the recognition of multiple scales and locations (Rosa et al. 2017).

However, so far, approaches to ES and scenario research that follow such recommendations remain scarce. To date, many ES assessments are aggregate assessments that continue to overlook the need to disaggregate by dimensions that are clearly relevant to distributional, recognitional and procedural equity (Suich et al. 2015; Cruz-Garcia et al. 2017; Mandle et al. 2020; Brück et al. 2022). Moreover, few ES studies are integrative and combine biophysical and social analyses, especially when it comes to valuation (Chan and Satterfield 2020). When analyzing ES in scenarios, the social component is often overlooked (Rosa et al. 2017; Felipe-Lucia et al. 2022), and scenario analyses that model both ecological and social variables are rare (Felipe-Lucia et al. 2022).

To address these gaps, we present here a generalized, social-ecological approach to support land use decision-making, and its application in a case study in the Global South. Our approach combines scenario planning with disaggregated ES analyses, in order to better address equity issues in the face of plausible trajectories of land use change (Jiren et al. 2020b), by (1) ensuring that the decision-making processes are more equitable (recognitional and procedural equity), and (2) analyzing the ES-related outcomes of land use change in terms of (distributional) equity. We follow a social-ecological approach, understanding social-ecological systems as interdependent and linked systems of people and nature, which are nested across scales (Fischer et al. 2015), and combining ecological and social data to evaluate multidimensional, disaggregated ES-related outcomes under different future scenarios. We qualify our approach as social-ecological, because ES, which are the focus of our analysis, are in themselves social-ecological phenomena at the core of the interactions between humans and nature, and ES analyses have been widely used as a proxy to understand social-ecological dynamics (Raudsepp-Hearne et al. 2010; Hamann et al. 2015). The bidirectional social-ecological interactions that some argue are key parts of social-ecological systems research (see, for example, Guerrero et al. 2018) are primarily captured via the use of participatory scenario planning that seeks to capture context-specific, social-ecological dynamics in order to develop feasible development pathways.

Our approach addresses distributional equity issues, by drawing out, in a spatially explicit way, the potential provision of ES and associated changes of socioeconomic outcomes for different beneficiary groups. The proposed approach also helps to address recognitional and procedural equity issues (Loos et al. 2022). In this way, we extend existing equity-related scenario analyses of ES (Felipe-Lucia et al. 2022; Neyret et al. 2023) by explicitly recognizing different dimensions of disaggregation, and by adding (1) the component of participatory scenario planning, (2) spatially explicit results of land use and land cover changes (LULC) and ES changes, and (3) a focus on the local population through the assessment of the values they ascribed to ES. Our approach can thus support researchers to provide knowledge on different future trajectories to local people and decision-makers in ways that are useful for planning for the future, while considering equity implications.

Our paper is structured as follows. First, we present a generalized, social-ecological approach to support land use decision-making, with six specific steps to follow, including a justification for each step and potential methods to use. As a second step, we present a case study for how to apply our general approach, in which we draw out the biophysical and socioeconomic implications of four future scenarios for local people and decision-makers in our study area in Ethiopia.

## A social-ecological approach to support equitable land use decision-making

Different dimensions of ES disaggregation (by beneficiary groups, value types, space; Brück et al. 2022) shed light on different equity issues (distributional, recognitional, procedural; Loos et al. 2022; Leach et al. 2018). Generally, the disaggregation by beneficiary groups is useful in identifying mainly distributional equity issues, but also power issues related to the provision and appropriation of ES (Daw et al. 2011; Felipe-Lucia et al. 2015; Martín-López et al. 2019). Connected to this, the broader social-ecological context of ES provision and appropriation, such as governance or the composition of stakeholders, shape who benefits in which ways, and can reveal important recognitional and procedural equity and power issues (Felipe-Lucia et al. 2015; Martín-López et al. 2019; Jiren et al. 2022). Disaggregation of value types (instrumental, relational and intrinsic values; IPBES 2022) and plural valuation recognize the plurality of values and worldviews and can contribute to more equitable and environmentally sustainable decisions (Arias-Arévalo et al. 2017; Zafra-Calvo et al. 2020; IPBES 2022). Spatial disaggregation can reveal how ES are provided and appropriated at different spatial scales, and can help reveal distributional equity issues (Hein et al. 2006; Liu et al. 2013; Schröter et al. 2018).

We propose six steps to generate and analyze scenarios of disaggregated, landscape-scale changes in land use as well as ES provision and appropriation (Table 1). These steps are intended to support more equitable decision-making in the context of land use change and ES management. They are (1) Set system boundaries and units of analysis for spatial (dis-)aggregation, (2) Develop narrative scenarios, (3) Translate scenarios into spatially explicit LULC maps, (4) Analyze biophysical changes related to ES, (5) Analyze socioeconomic changes related to ES, and (6) Communicate results for decision-making.

**Table 1:** Six steps of a generalized, social-ecological approach to support land use decision-making, including a general description of each step, as well as potentially related equity issues and disaggregation dimensions. We consider distributional, recognitional, and procedural equity issues; we consider disaggregation of ES provision and appropriation by beneficiary groups, value types, and space

Step	General description (what to do and why)	Equity issues and disaggregation dimensions
(1) Set system boundaries and units of analysis for spatial (dis-) aggregation	Identify the landscape and set the system boundaries; consider village or municipality level as a key unit of analysis; use clustering techniques to identify social-ecological archetypes	Equity: distributional, recognitional Disaggregation: beneficiary groups, space
(2) Develop (narrative) scenarios	Define plausible future trajectories through participatory scenario planning; this is the qualitative knowledge basis for quantitative modeling	Equity: recognitional, procedural Disaggregation: beneficiary groups, space, value types
(3) Translate scenarios into spatially explicit LULC maps	Use explicit rules to translate narratives into quantitative assessments of land use change (e.g., agent-based modeling, fuzzy cognitive maps, or Bayesian networks); this is the first step for further quantitative analyses of biophysical and socioeconomic implications	Equity: distributional Disaggregation: space
(4) Analyze biophysical ES changes	Based on LULC data; frequently used methods include the use of causal relationships, extrapolation of ES values from primary data, and regression models; this is the basis for subsequent socioeconomic analyses	Equity: distributional, recognitional Disaggregation: beneficiary groups, space
(5) Analyze socioeconomic ES changes	Further analyze ES provision and appropriation along social, political, economic aspects, based on range of methods for disaggregated ES and stakeholder analysis (e.g., plural valuation, social network analysis); useful for identifying and acknowledging equity and power issues, plurality of values and worldviews	Equity: distributional, recognitional, procedural Disaggregation: beneficiary groups, space, value types
(6) Communicate results for decision-making	Re-arrange biophysical and socioeconomic results depending on the related policy question; results should be accessible for locals to develop spatially differentiated polices or strategies	Equity: distributional, recognitional, procedural Disaggregation: beneficiary groups, space, value types

### Step 1: Set system boundaries and units of analysis for spatial (dis-)aggregation

The first step is to identify the landscape and set the system boundaries. In this way, following Wu (2013), the approach represents a place-based assessment at the landscape scale in a spatially explicit manner, which helps to understand and improve the dynamic relationship between ES and human well-being in changing landscapes. Once the landscape is identified, choosing meaningful units of analysis is essential for further assessments of land use and ES at different levels of spatial (dis-) aggregation under the scenarios. The village or municipality level can often be a useful unit of analysis, because social and ecological data are often available at this level, and it is usually also the finest scale at which land use decision-making takes place (Hanspach et al. 2016; Martín-López et al. 2017; Pacheco-Romero et al. 2022). Based on the unit of analysis, clustering techniques can then be used to define social-ecological archetypes (Sietz et al. 2019; Rocha et al. 2020; Pacheco-Romero et al. 2021). Archetypes retain the richness of case studies, while identifying context-sensitive, generalizable patterns that can help to support evidence-based decision-making (Oberlack et al. 2019). Aggregation into archetypes is especially useful for social-ecological systems, where recurrent patterns of social and ecological phenomena occur across the study area (i.e., relatively homogeneous spatial units that share similar social-ecological characteristics or interactions; Martín-López et al. 2017). The use of such archetypes can help to work with the social-ecological complexity of the study area, and therefore to conduct further assessments, interpret the data, and make results accessible and easier to communicate. This step can address distributional and recognitional equity, mainly through spatial disaggregation (Table 1).

### Step 2: Develop narrative scenarios

The second step is to develop scenario narratives for the chosen landscape through participatory scenario planning (Peterson et al. 2003; Oteros-Rozas et al. 2015). This method engages multiple perspectives of diverse stakeholders, which can potentially reduce power asymmetries and increase the legitimacy of the results (if applied carefully), and helps to explore uncertain, but plausible future land use changes (Oteros-Rozas et al. 2015; Jiren et al. 2020b). Participatory narrative scenarios seek to develop plausible LULC change pathways based on qualitative knowledge regarding current and potential future dominant social-ecological system dynamics driving such changes (e.g., key ES relevant to stakeholders, key variables, causal mechanisms and feedbacks that shape overall social-ecological dynamics; Oteros-Rozas et al. 2015; Jiren et al. 2020b; Duguma et al. 2022). While such participatory narrative scenarios do not directly address equity outcomes, the process of developing such scenarios may be an important first step in recognizing issues related to recognitional and procedural equity, and resulting scenario narratives may refer to multiple disaggregation dimensions with regard to ES provision and appropriation.

### Step 3: Translate scenarios into spatially explicit LULC maps

In the third step, the scenario narratives are translated into spatially explicit, quantitative assessments of land use change and LULC maps. A range of methods can be used here, for example, agent-based modeling, fuzzy cognitive maps, or Bayesian networks (Mallampalli et al. 2016). The resulting LULC maps can then be used to quantify, model and map ES (Burkhard 2017; Crossmann 2017; Vihervaara et al. 2017). Producing explicit LULC maps facilitates the comparison of the different scenarios in a spatially explicit way, and provides a sound basis for further quantitative analyses of biophysical and socioeconomic implications (Duguma et al. 2022). However, such translations should be undertaken with care and with attention to biases, and, if possible, in a participatory way, especially since translations of narratives into LULC rules can be fraught with assumptions. This step addresses distributional equity through spatial disaggregation (Table 1).

### Step 4: Analyze biophysical ES changes

Based on the LULC maps, biophysical ES changes can be analyzed. The ES to analyze should best be chosen based on their relevance to local stakeholders, and, if available, using previous research of the study area (Manlosa et al. 2019; Jiren et al. 2020b). The selection of appropriate methods depends on, among others, overall study aims, types of services to map, accuracy required, expected impact in decision-making, mapping skills, and time and data availability (Malinga et al. 2015; Palomo et al. 2017). Frequently used sources of information for ES provision mapping include land cover variables, topographical information and spectral vegetation indices, and frequently used methods include the use of well-known causal relationships between environmental variables, extrapolation of ES values from primary data, and regression models (Martínez-Harms and Balvanera 2012; Burkhard and Maes 2017; Alexis Akakpo et al. 2023). As the basis for all subsequent analyses of socioeconomic changes, biophysical changes in ES provision need to be made spatially explicit. In terms of equity dimensions, this step mainly addresses distributional equity through spatial disaggregation, but also recognitional equity if ES are selected based on local relevance.

### Step 5: Analyze socioeconomic ES changes

The understanding of biophysical ES changes generated in steps 3 and 4 is then the basis for exploring the social, ecological political, and economic changes that occur under the different scenarios. The specific changes considered will be very context-dependent, but should be related to the analysis of ES provision and appropriation along the dimensions of beneficiary groups, value types, and space (Brück et al. 2022). For example, through further analysis of ES provision (e.g., who specializes on generating which ES?), analysis of governance aspects, or ES valuation based on plural values (for methodological details in the context of our case study, see section "Study area and methods"). Such analyses can be useful for identifying and acknowledging equity and power issues as well as for working with a plurality of values and worldviews (Reed et al. 2009; Brück et al. 2022). This step can help to address distributional, recognitional and procedural equity issues, through the disaggregation along multiple dimensions.

### Step 6: Communicate results for decision-making

To communicate the biophysical and socioeconomic results, they should be rearranged and summarized according to specific policy questions. Results should be made accessible for local people and decision-makers to be useful for the development of spatially differentiated polices or strategies to mitigate or encourage specific land use change trajectories (Brück et al. 2022; Duguma et al. 2022). Visualization of data and results is very important and should consider trade-offs between context-specificity, generalization, and communicability (Harold et al. 2016; Magliocca et al. 2018; Böttinger et al. 2020; Metze 2020). The operationalization of biophysical and socioeconomic results is the last step toward better recognition of distributional, recognitional, and procedural equity issues in land use decision-making, through the consideration of multiple dimensions of disaggregation.

## Case study in southwestern Ethiopia

We applied the general approach outlined above to a case study in southwestern Ethiopia (Table 2). We conducted a disaggregated analysis of implications of LULC and connected ES changes under four future scenarios, considering different equity issues and disaggregation dimensions. Many of the detailed steps of our overall analysis have been published as stand-alone papers in the past; below, we bring the findings together following the six-step process outlined above. Readers are asked to consult the referenced papers for additional methodological details for the prior work already published.

**Table 2:** Application of our generalized, social-ecological approach to the case study, and related equity issues and disaggregation dimensions for each of six steps

Step	Application in Ethiopian case study	Equity issues and disaggregation dimensions in the case study
(1) Set system boundaries and units of analysis for spatial (dis-) aggregation	66 kebeles (smallest administrative units in Ethiopia) within a landscape spanning three districts were identified as meaningful units of analysis, and were clustered into four social-ecological archetypes	Equity: Kebeles as units of analysis and archetypes as a means of aggregation are the basis to make distributional differences explicit (distributional); four different archetypes with differing values and social-ecological contexts (recognitional) Disaggregation: Kebeles of each archetype are in specific locations (space); each archetype is characterized by a specific social-ecological context, and hence represents different groups of beneficiaries (beneficiary groups)
(2) Develop (narrative) scenarios	Participatory scenario planning for 2040 with over 30 groups of local people and stakeholders	Equity: Local stakeholder groups and their perceptions about plausible landscape change are acknowledged (recognitional); knowledge of different stakeholder groups is incorporated into scenarios, which might be used for future policy making (procedural) Disaggregation: Scenario narratives mention all disaggregation dimensions to a certain extent (beneficiary groups, value types, space)
(3) Translate scenarios into spatially explicit LULC maps	Baseline LULC maps were based on satellite imagery, plus rules for how to modify the baseline map under each scenario, based on the narratives; proximity-based InVEST scenario generator was used to create LULC maps for the four scenarios	Equity/Disaggregation: Spatial distribution of LULC across the landscape is mapped explicitly at kebele and archetype level, for baseline and scenarios (distributional, space)
(4) Analyze biophysical ES changes	Modeling of potential per capita provision of 11 locally important ES, based on the LULC maps derived in step 2, for each kebele, for the baseline as well as the four scenarios	Equity: Choice of ES based on needs of and relevance for local smallholders (recognitional); Spatial distribution of potential provision of ES is made explicit at kebele and archetype level, for baseline and scenarios (distributional) Disaggregation: Spatial distribution of potential provision of ES is made explicit at kebele and archetype level, for baseline and scenarios (space, beneficiary groups)
(5) Analyze socioeconomic ES changes	Analysis of three different aspects: degree of ES specialization (based on ES potential provision); importance of four different value types (based on ES potential provision and values ascribed to ES); presence of stakeholder groups with specific interests in local ES	Equity: Degree of specialization as well as value types are made spatially explicit at kebele and archetype level (distributional); beneficiary groups in each kebele archetype and the different value types that they ascribe to ES are recognized (recognitional); analysis of stakeholder presence is a first step toward their consideration in land use decision-making processes (procedural) Disaggregation: Disaggregated analysis of specialization and values by kebele and archetypes (beneficiary groups, value types, space)
(6) Communicate results for decision-making	Results were re-arranged to understand how well kebeles of each archetype do on average (with regards to ES potential provision and value types ascribed to ES) at the baseline and in the scenarios, and how variable the outcomes are	Equity: Mean outcomes and their variability are explicit at the archetype level (distributional) Disaggregation: Results at the baseline and for each scenario are (dis-) aggregated to the archetype level (beneficiary groups, space)

### Study area and methods

For Step 1 (Set system boundaries and units of analysis for spatial (dis-)aggregation), we focused on a landscape in southwestern Ethiopia, which is undergoing rapid social-ecological change due to different social, demographic, economic, environmental, technological, political and governance drivers, including population growth, land use and climate change (Jiren et al. 2020b). The study area consists of three woredas (districts), in Jimma Zone, Oromia Region, Ethiopia, namely Gera, Gumay, and Setema woreda, which comprise 66 kebeles (the smallest administrative units in Ethiopia). Kebeles in the study area measure on average 30 km^2^ and have an average population of 4000 inhabitants. The landscape is characterized by a mosaic of arable land and pastures, interspersed by woody vegetation, and moist evergreen Afromontane forest (which amounts to roughly 50% of the current landscape), and is a recognized biodiversity hotspot (Mittermeier et al. 2011; Hylander et al. 2013; Beenhouwer et al. 2016). Local smallholder farmers depend on nature, and ES are locally important for subsistence, income generation and culture (Shumi et al. 2019; Schultner et al. 2021). Livelihood strategies have traditionally been diversified and subsistence-oriented (Manlosa et al. 2019). However, specialization and market integration are strongly encouraged by the government, and many stakeholders expect or favor such developments (Federal Democratic Republic of Ethiopia, National Planning Commission 2016; Jiren et al. 2020a, 2020b). Over the past decades, production has begun to shift from subsistence to marketed crops (Schultner et al. 2021). Government, non-governmental, private and community-based organizations are active in the landscape (Jiren et al. 2022). The governance related to land use and ES management is often strongly hierarchical and dominated by government administrative organizations (Jiren et al. 2018, 2022). We chose the kebele level as our minimum unit of analysis, because it is meaningful for local people and decision-makers, and both ecological and social data were available at that level. For more meaningful interpretation and communication of the results, we clustered the 66 kebeles in our study area into four social-ecological, system-specific archetypes, based on nine (current) ecological and social variables, using hierarchical clustering (see Duguma et al. 2022 for further methodological details).

For Step 2 (Develop narrative scenarios), we conducted participatory scenario planning with over 30 groups of local people and stakeholders, using multiple rounds of workshops between 2015 and 2019. We co-generated four scenario storylines that plausibly narrate how the landscape might develop until 2040 (see Fischer et al. 2018 and Jiren et al. 2020b for further methodological details).

For Step 3 (Translate scenarios into spatially explicit LULC), we developed a baseline LULC map based on satellite imagery, and, drawing on the previously developed narrative scenarios, defined rules for how to modify the baseline map under each scenario. We then used the proximity-based scenario generator of the InVEST software to create LULC maps of the four scenarios (see Duguma et al. 2022 for further methodological details).

For Step 4 (Analyze biophysical ES changes), we selected 11 locally relevant ES (see section on ES selection in the Supplementary Information for details), namely beef, biodiversity, cattle, firewood, honey, khat, maize, plantation coffee, semi-forest coffee, sorghum, and teff. We modeled the potential provision of each ES per capita for each kebele at the baseline and in the four scenarios, mainly through regression models, based on the LULC data derived in step 2, as well as additional ecological and social data (see section on ES potential provision in the Supplementary Information for further methodological details). We chose per capita measures of ES, because they highlight the amounts that could potentially be appropriated by local people (Spangenberg et al. 2014). Then, the results were re-aggregated and averaged across kebeles for the entire study area, and for each of the four social-ecological archetypes that resulted from step 3, both for the baseline and the four scenarios. The scenarios were assessed in terms of changes relative to the current (baseline) situation. Here it is important to note that our analysis does not account for potential inequalities among the beneficiary groups in the baseline. Therefore, the analysis can provide useful information regarding potential “winners and losers” under different development scenarios, but not regarding ‘ideal’ (e.g., equitable) distribution of ES across the study area.

For Step 5 (Analyze socioeconomic ES changes), we chose to analyze three different socioeconomic aspects, namely the degree of ES specialization (which comes with opportunities and risks, e.g., through increased agricultural yield, but also through increased vulnerability to ecological and economic shocks, Abson 2019), the types of values ascribed to ES by local people, and the presence of different stakeholder groups in the landscape (governance aspects around ES can have important equity and power implications; Felipe-Lucia et al. 2015). For *ES specialization*, we defined that a kebele was more specialized if its ES provision was more concentrated across ES. We calculated the degree of ES specialization through Simpson’s index based on adjusted ES potential provision data for each kebele (divided by total kebele area, logarithmic transformation, min–max scaled), at the baseline and for the four scenarios (see section on ES specialization in the Supplementary Information for details). In contrast to the potential provision results above, we adjusted here for total kebele area instead of population, because specialization measures changes with a focus on the landscape level. To analyze the *types of values ascribed to ES* by local people, we used per capita ES provision data (again, highlighting the perspective of local people), and data on four different value types (direct use, exchange, relational, intrinsic) ascribed to ES at the baseline (derived from surveys of 164 local participants). We calculated the importance of the four value types for each kebele, at the baseline and for the four scenarios (see section on value types in the Supplementary Information for details). Finally, we analyzed the *presence of different stakeholder groups with specific interests in local ES* in each scenario and compared them to the baseline, based on stakeholder interviews with the help of space-for-time substitution (selecting four existing landscapes nearby as proxies representing the types of changes described in the four scenarios; see Jiren et al. 2022 for further methodological details). As in step 4, the results were re-aggregated and averaged for the entire study area and for each social-ecological archetype (except for the presence of different stakeholder groups).

Finally, for step 6 (Communicate results for decision-making), we took the resulting kebele level data across the baseline and the four scenarios, separately for each aspect of steps 4 and 5 (provision of each ES, specialization, and importance of value types), and split them into seven equally sized groups (only three for stakeholder presence). This resulted in seven categories for each aspect, ranging from “extremely low” to “extremely high”, which indicated the relative level of the aspect in a kebele in comparison to all other kebele results across the baseline and the scenarios. We re-aggregated our results further by summarizing ES potential provision and the value types ascribed to the ES. Specifically, we calculated the mean relative level and its standard deviation across the provision of all ES and the value types for each archetype at the baseline and for each scenario. First of all, this helped to understand how well each archetype did on average at the baseline and in the scenarios, and facilitated an overarching comparison between archetypes (who is better or worse off?). In addition, it allowed to understand how variable outcomes for each archetype were across scenarios, and hence to assess the resilience of each archetype.

### Results

**Fig. 1:**
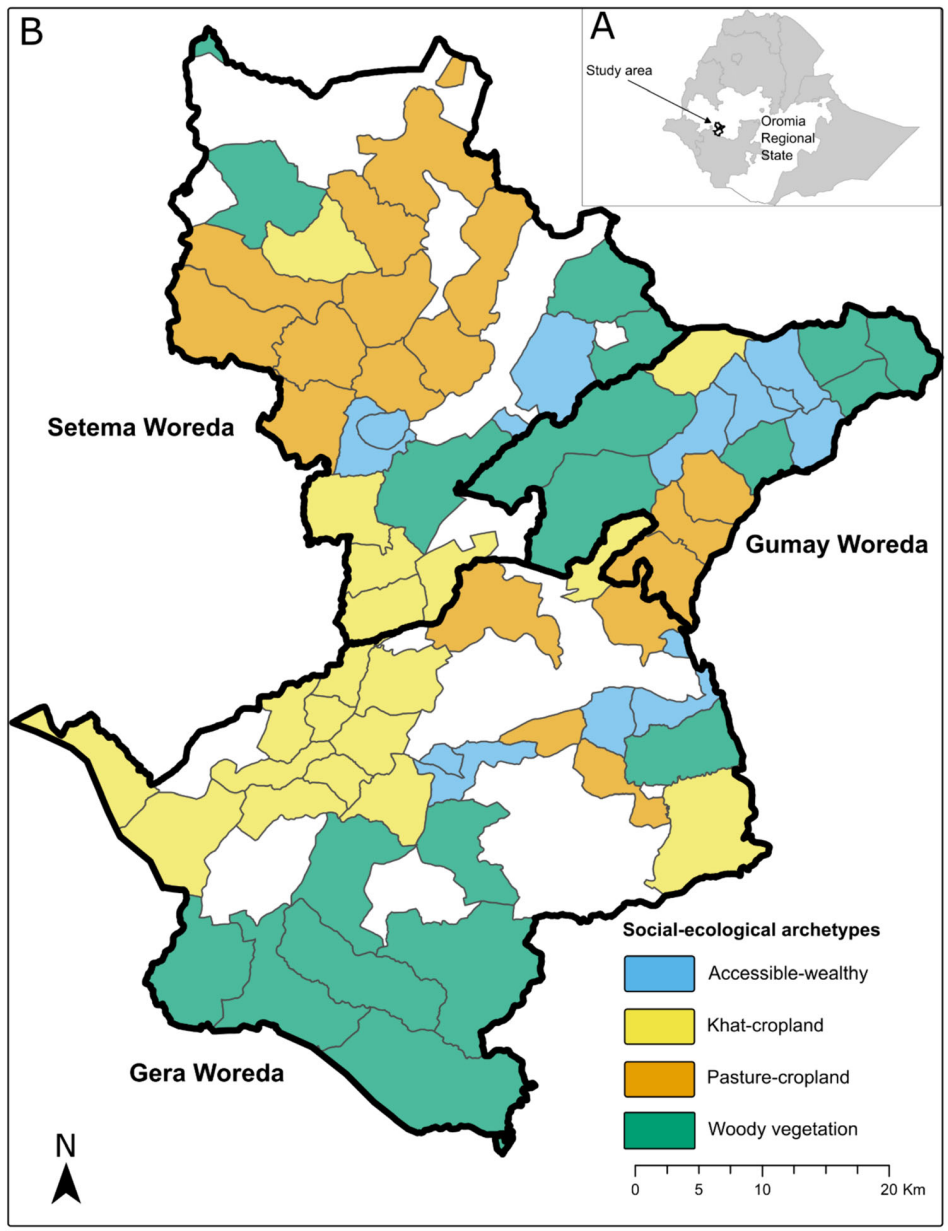
Map of the study area in Oromia regional state in Ethiopia (A). The 66 kebeles (smallest administrative units in Ethiopia) belong to one of four social-ecological archetypes in three different woredas (districts) (B). Kebeles without color are forest kebeles and excluded from the analysis

In step 1, based on their baseline characteristics, the 66 kebeles in our study area were clustered into four social-ecological archetypes, namely the accessible-wealthy, the khat-cropland, the pasture-cropland and the woody vegetation archetype (see Fig. 1 for a map, Table 3 for short descriptions, Table S1 for a list of all kebeles and their respective archetype; Duguma et al. 2022). Each of these archetypes represented a specific social-ecological context, and hence different groups of beneficiaries, in a spatially explicit way.

**Table 3:** Social-ecological kebele archetypes and narrative scenarios (adapted from Duguma et al. 2022 and Jiren et al. 2020b)

Social-ecological archetypes	
Accessible-wealthy	12 kebeles with large extents of eucalyptus plantations, relatively accessible and wealthy
Khat-cropland	19 kebeles with distinctly high availability of khat and arable land, located at higher altitudes, with low coffee forest availability and the lowest wealth index
Pasture-cropland	17 kebeles with high availability of pasture and arable land, with the lowest cover of woody vegetation and low levels of coffee forest, khat, and eucalyptus
Woody vegetation	18 kebeles with high extent of woody vegetation cover, high coffee forest availability and high importance of honey production, relatively remote

Narrative scenarios	
Gain over grain: Local cash crops	The scenario was characterized by intensive cash crop production, with large plots of intensively managed coffee, interspersed with khat and tree plantations, which are sold at local, national, and global markets
Coffee and conservation: Biosphere reserve	In the scenario, a biosphere reserve was established, and the landscape consisted of a mosaic of diversified farmland and forest patches, which combined nature conservation, sustainable agriculture, ecological coffee production, and tourism opportunities
Mining green gold: Coffee investors	In the scenario, communal lands and forests were transferred to domestic and foreign large-scale coffee investors for the production and export of commercial and specialized coffee
Food first: Intensive farming and forest protection	The scenario was characterized by industrialized agriculture involving high-yielding varieties and agrochemical inputs, and strictly protected patches of natural forest

**Fig. 2:**
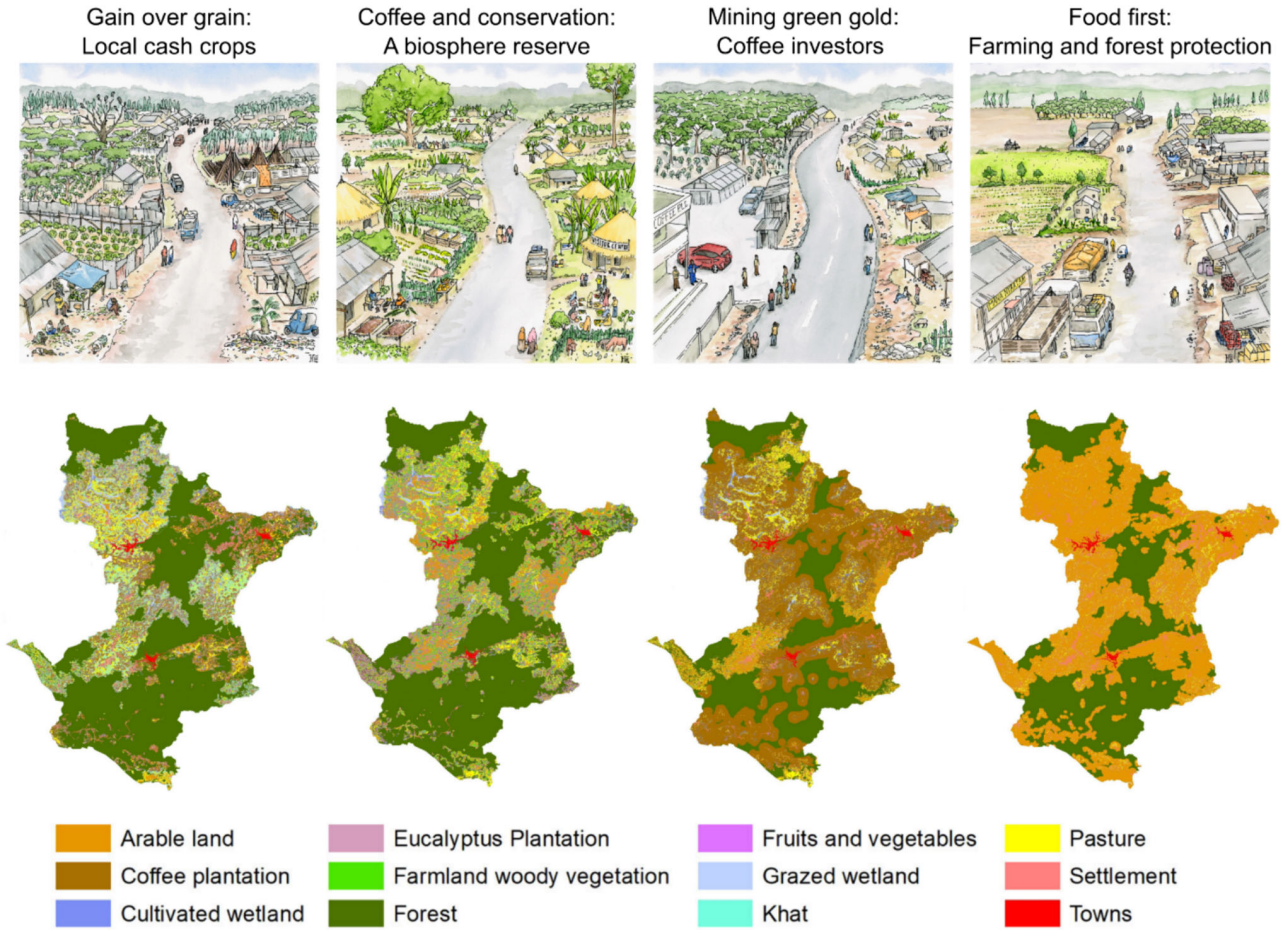
Visual representation of the key features in terms of landscape features and composition in a village, and LULC maps for four future scenarios: (1) Gain over grain: Local cash crops; (2) Mining green gold: Coffee investors; (3) Coffee and conservation: A biosphere reserve; and (4) Food first: Intensive farming and forest protection (adjusted from Duguma et al. 2022 and Jiren et al. 2020b)

In step 2 and 3, the participatory scenario planning exercise, which recognized knowledge and perceptions of different local stakeholder groups, resulted in four scenario narratives of how the trajectory of the landscape might change by 2040, which were also translated into spatially explicit LULC maps (see Fig. 2 for visual representations and LULC maps, Table 3 for short descriptions; Jiren et al. 2020b; Duguma et al. 2022). The four scenarios were named “Gain over grain: Local cash crops”, “Coffee and conservation: Biosphere reserve”, “Mining green gold: Coffee investors”, and “Food first: Intensive farming and forest protection”. Generally, the first two scenarios were characterized by integrated land uses, whereas the last two were characterized by segregated land uses and intensification.

**Fig. 3:**
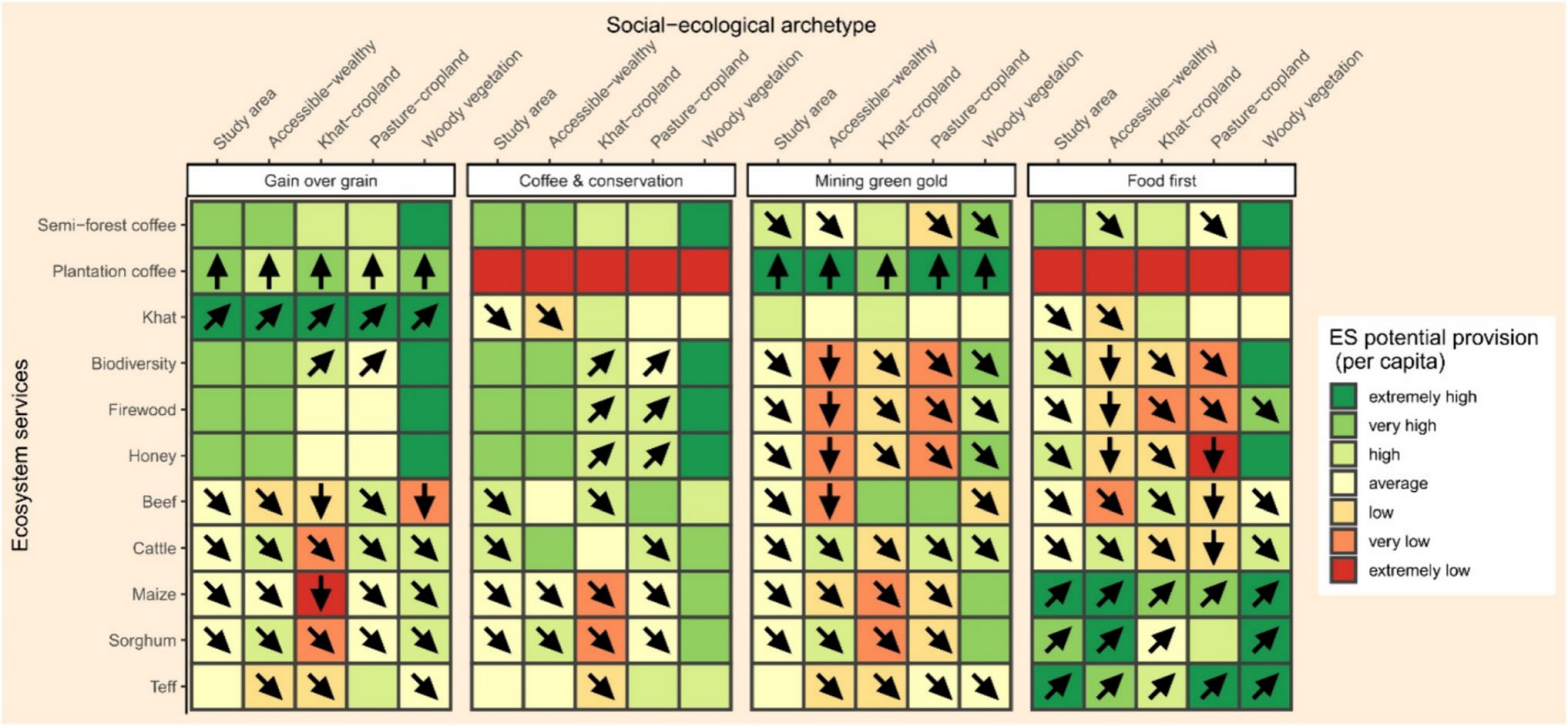
Relative levels of per capita potential provision of 11 locally important ES under four scenarios, for the entire study area and for each social-ecological archetype. To obtain the seven levels, ranging from “extremely high” to “extremely low”, kebele level data across the baseline and the scenarios for each ES were split into seven equally sized groups. Arrows indicate changes from the baseline: upward arrow indicates an increase in the relative level of ES potential provision (+ 6 to + 4), diagonal upward arrow indicates a moderate increase (+ 3 to + 1), no arrow indicates no change, diagonal downward arrow indicates moderate decrease (-1 or -2), and downward arrow indicates decrease (-3 or -4). For example, under the “Gain over grain” scenario, the level of potential provision of maize per capita for the khat-cropland group is extremely low and has decreased compared to the baseline

In step 4, the level of ES potential provision per capita not only differed between the four scenarios, but also between archetypes (Fig. 3; for a more detailed description of the results, see Supplementary Information). For the study area on average (first column in each scenario block), the “Gain over grain” scenario, compared to the baseline, was mostly characterized by increases in the level of potential provision per capita of plantation coffee and khat, at the expense of livestock and cereal crops. Under the “Coffee and conservation” scenario, potential provision of most ES remained the same compared to the baseline, or only changed slightly. The “Mining green gold” scenario showed an increase in plantation coffee, and the “Food first” scenario showed increases in all cereal crops, whereas many other ES decreased.

In contrast to these overall tendencies, the additional analysis by archetype revealed differences in the spatial distribution of potential provision of ES. Whereas observed changes in the archetypes were never opposite (e.g., if an ES decreased under one scenario for the study area on average, we also saw either no change or a decrease for each archetype), potential provision per capita often showed contrasting levels for certain ES under the same scenario, e.g., under the “Gain over grain” scenario, maize was extremely low for the khat-cropland archetype, but high for the woody vegetation archetype. Similarly, under the “Mining green gold” scenario, despite the increase in plantation coffee, the woody vegetation archetype still showed high or very high levels for woody vegetation related ES (semi-forest coffee, biodiversity, firewood, honey), whereas all other archetypes showed lower levels for these ES. Compared to the study area average, the woody vegetation archetype had higher (or the same) potential provision per capita for almost all ES (except for khat and beef) across the baseline and all scenarios. By contrast, the khat-cropland archetype had lower (or the same) potential provision level for almost all ES (except for khat and beef).

**Fig. 4:**
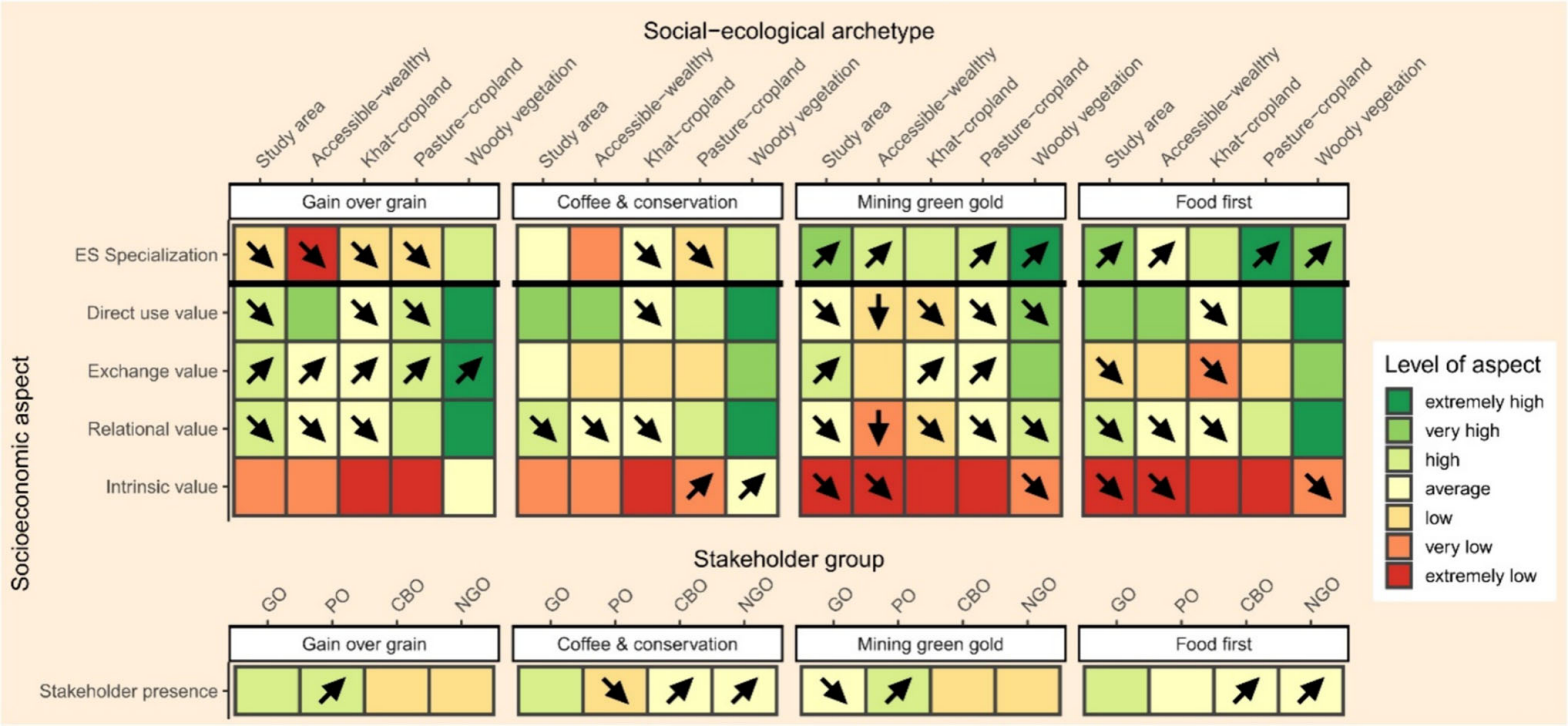
Relative levels of different socioeconomic aspects under four scenarios, for the entire study area and for each social-ecological archetype. To obtain the seven levels, ranging from “extremely high” to “extremely low, kebele level data across the baseline and the scenarios for ES specialization and all value types were split into seven equally sized groups. Stakeholder presence measures the proportions of stakeholder groups at the study area level (GOs = Governmental organizations, POs = Private organizations, CBOs = Community-based organizations, NGOs = Non-governmental organizations), and was only split into three equally sized groups. Arrows indicate changes from the baseline: upward arrow indicates an increase in the relative level (+ 6 to + 4), diagonal upward arrow indicates a moderate increase (+ 3 to + 1), no arrow indicates no change, diagonal downward arrow indicates moderate decrease (− 1 or − 2), and downward arrow indicates decrease (− 3 or − 4). For example, under the “Mining green gold” scenario, direct use value for the woody vegetation archetype is high, but has decreased compared to the baseline

In step 5, the three socioeconomic aspects (ES specialization, value types, stakeholder presence) differed between the four scenarios, but also between archetypes (Fig. 4; for a more detailed description of the results, see Supplementary Information; for boxplots of specialization and values results see Figs. S3–S6). For the study area on average (first column in each scenario block), ES specialization decreased or remained the same under the two integrated land use scenarios (“Gain over grain” and “Coffee and conservation”), and increased under the two intensification scenarios (“Mining green gold” and “Food first”). For the value types, the “Gain over grain” scenario saw an increase in exchange value, but decreases in direct use and relational value, whereas the “Coffee and conservation” scenario saw almost no changes. The two intensification scenarios were characterized by decreases in almost all value types. In the “Gain over grain” and the “Mining green gold” scenarios, the proportion of private organizations present in the landscape increased, whereas the proportion of community-based and non-governmental organizations increased for the other two scenarios.

In addition, analyzing the socioeconomic aspects by archetype recognized and revealed differences between these four beneficiary groups. Whereas observed changes in the archetypes were never opposite (but instead went in the same direction for all archetypes), socioeconomic aspects showed sometimes contrasting levels under the same scenario, e.g., under the “Mining green gold” scenario, relational value was low for the khat-cropland archetype, but high for the woody vegetation archetype. Again, the woody vegetation archetype and the khat-cropland archetype showed contrasting results. Generally, compared to the study area average, the woody vegetation archetype showed mostly higher-than-average levels for specialization, and higher levels for all value types. By contrast, the khat-cropland archetype had mostly lower specialization levels than the study area average, and all value types were consistently lower or the same as average.

**Fig. 5:**
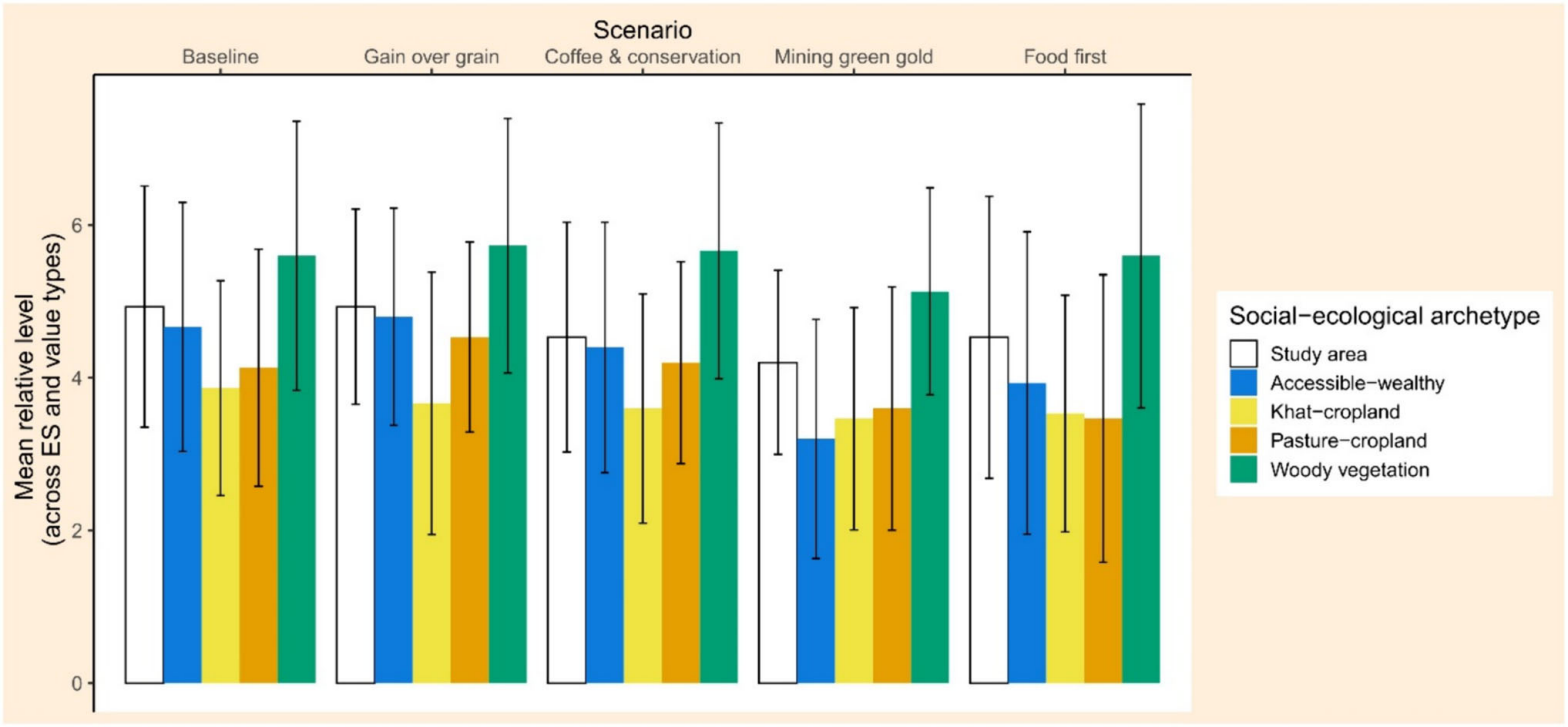
Bar plots of mean relative levels across ES provision and the value types ascribed to them at the baseline and under the four scenarios, for the study area and four social-ecological kebele archetypes, with error bars (based on standard deviations). ES provision is the potential per capita provision of 11 locally important ES; value types include direct use, exchange, relational, and intrinsic value. The heights of the bar plots indicate the mean relative level across ES provision and value types; the error bars indicate the variability of relative levels across ES and value types, based on the standard deviation

For step 6, at a further level of aggregation (Fig. 5), the woody vegetation archetype was again better off than the other archetypes, and showed comparatively high mean relative levels of ES provision and ascribed values. In contrast, the khat-cropland archetype was the relatively worst-off archetype, with comparatively low mean levels at the baseline and under all scenarios. Both the pasture-cropland and the accessible-wealthy archetype did well enough under the two scenarios with integrated land use (“Gain over grain” and “Coffee and conservation”), but did worse under the two intensification scenarios (“Mining green gold” and “Food first”).

## Discussion and outlook

We proposed a step-by-step approach to facilitate more equitable land use decision-making. Through the recognition of different beneficiary groups, value types and spatial locations, and by bringing together ecological and social data in scenario planning with ES as key response variables (Rosa et al. 2017; Chan and Satterfield 2020), we showed how a more equitable decision-making process can be ensured, made equity-related effects of land use change explicit (Schlosberg 2007; Felipe-Lucia et al. 2022), and hence increased the decision relevance of landscape-scale scenarios of land use and ES change (Mandle et al. 2020).

Scenario planning is a useful tool to anticipate effects of land use change in complex social-ecological systems, and, if implemented in a participatory way, can give agency to local people when thinking about potential trajectories of their landscape (Oteros-Rozas et al. 2015; Jiren et al. 2020b). However, the development of scenario narratives on their own may be insufficient to fully understand equity-related effects of land use change, and to meaningfully guide decision-making, and hence needs to be complemented by further analysis of ES provision and appropriation (Rosa et al. 2017; Felipe-Lucia et al. 2022).

Disaggregated analyses of ES provision and appropriation along three dimensions, namely beneficiary groups, value types, and space, can inform more equitable and sustainable decision-making, but such analyses remain rare (Bennett et al. 2015; Mandle et al. 2020). Successful case study examples, similar to our case study, disaggregate ES along several dimensions, generating equity-relevant insights that would not have been possible under an aggregated approach (Dawson and Martin 2015; Arias-Arévalo et al. 2017; Dorresteijn et al. 2017). However, Mandle et al. (2020) found that only a third (31%) of ES assessments disaggregated spatially, and even fewer assessments (7%) disaggregated by beneficiary groups. Our proposed approach opens new opportunities to explicitly recognize different dimensions of disaggregation, namely beneficiary groups, value types and space. Throughout all steps at least one of these dimensions was recognized, and in our application, we disaggregated along these three dimensions in multiple ways: we derived spatially explicit social-ecological archetypes, which represented different beneficiary groups; we recognized different stakeholder groups in the development of scenario narratives; we identified value types as key socioeconomic variables; and we made our results with regard to LULC and ES changes spatially explicit at the kebele as well as the archetype level.

Our social-ecological approach takes ES as key scenario variables, bringing together different types of data and methods for a disaggregated assessment of plausible future land use change. Throughout all steps of the proposed approach, both ecological and social data were required and generated. The application to our case study showed how ecological and social data can successfully be combined to evaluate and present multidimensional, disaggregated ES-related outcomes under different future scenarios. For example, ecological and geographical data were used to model ES potential provision, whereas social data were the basis to assess the value types ascribed to ES, or stakeholder presence. We also used different natural and social science methods, such as ecological field surveys, remote sensing, stakeholder interviews and household surveys. Such combination of ecological and social data in ES and scenario analysis has been recommended in the past (Fischer et al. 2017; Mandle et al. 2020; Felipe-Lucia et al. 2022), and has been applied in a range of other case studies, in order to assess equity and sustainability implications of land use change (Pacheco-Romero et al. 2021; Felipe-Lucia et al. 2022; Neyret et al. 2023).

Through the recognition of different beneficiary groups, value types and spatial locations, and by combining ecological and social data, our approach helped to conduct equity-focused analyses of ES under different scenarios. All steps of our proposed approach facilitated the consideration of distributional, recognitional, and procedural equity issues related to ES. In our case study, we drew out explicitly the equity implications of land use and related ES changes under four plausible trajectories and thus highlighted winners and losers in terms of biophysical and socioeconomic changes under the different scenarios. Generally, kebeles in the woody vegetation archetype were mostly better off than the other archetypes: they had higher (or the same) potential provision per capita for almost all ES across the baseline and all scenarios, and higher-than-average levels for all value types. However, they showed mostly higher-than-average levels for specialization (specialization may increase vulnerability to ecological and economic shocks due to decreased multifunctionality and resilience, Abson 2019). By contrast, kebeles in the khat-cropland archetype were comparatively worse off: they had lower (or the same) potential provision levels for almost all ES, and all value types were consistently lower or the same as average, but specialization levels were mostly same or lower than the study area average. The other two archetypes were sometimes better and sometimes worse off than the study area average, but they did generally worse under the two intensification scenarios (“Mining green gold” and “Food first”). We also incorporated recognitional and procedural equity in the decision-making process through the participatory approach to scenario planning, the recognition of the different kebele archetypes with differing values and social-ecological contexts, and the analysis of stakeholder presence. Similarly, Felipe-Lucia et al. (2022) focused on procedural and distributional equity, and Neyret et al. (2023) on distributional equity related to ES under different landscape management scenarios. We further added to these approaches the component of participatory scenario planning, spatially explicit results of LULC and ES changes, and a focus on the local population through the assessment of the values they ascribed to ES.

Overall, our case study revealed equity-related insights which would not have been derived from a simple aggregated ES assessment. What is more, our approach helped to improve recognitional and procedural equity in the process of decision-making. Therefore, future strategy development by local smallholders and decision-makers needs to be context-specific and inclusive, in order to appropriately mitigate and adapt to future changes. We recommend the application of our approach in other contexts, especially in the Global South, where, similarly to our case study region, people are often closely dependent on nature and also especially vulnerable to change. However, as a drawback, the application of our approach requires either existing knowledge of the case study region, or enough resources to obtain the required ecological and social data. Depending on the specific case study, the six steps we proposed can be used in a flexible way and be modified, for example through broader application of space-for-time substitution to obtain explicit social data for each future scenario, or by additionally including other-than-local stakeholders in the values assessment.

## Supplementary Information

Below is the link to the electronic supplementary material.

Supplementary file1 (PDF 623 KB)

## Data Availability

The data that support the findings of this study are available at 10.48548/pubdata-150.
