# Supplementary material for: A social-ecological approach to support equitable land use decision-making
Source: Ambio. 2024 Aug 2;53(12):1752–67. doi: 10.1007/s13280-024-02056-x (PMC11568087; doi:10.1007/s13280-024-02056-x)
Supplement: Supplementary file 1 — Supplementary file1 (PDF 623 KB) [file 13280_2024_2056_MOESM1_ESM.pdf]

Ambio

Supplementary Information

This supplementary information has not been peer reviewed.

Title: **A social-ecological approach to support equitable land use decision-making**

Methods

R version and packages

All analyses were conducted with the statistical computing language R (R Core Team 2022), R version 4.3.0, using the following R packages: “diverse” (Guevara et al. 2016), “ggpubr” (Kassambara 2020), “sjPlot” (Lüdecke 2022), “performance” (Lüdecke et al. 2021), “sf” (Pebesma 2018), “bestNormalize” (Peterson 2021), “tidyverse” (Wickham et al. 2019), “readxl” (Wickham and Bryan 2023), “ordinal” (Christensen 2022), “glmmTMB” (Brooks et al. 2017).

ES selection

We selected 11 locally important ES, based on previous research, insights from the literature and their relevance in the scenarios. Biodiversity is a supporting ES, which is locally relevant, but also of global importance (Beenhouwer et al. 2016; Duguma et al. 2023). The cereal crops maize (*Zea mays*), sorghum (*Sorghum bicolor*) and teff (*Eragrostis tef*) are the main food crops (Manlosa et al. 2019). Households usually own a small number of livestock, and cattle are used as draft animals and also considered a valuable capital asset (Manlosa, Schultner, Dorresteijn, & Fischer, 2019). We distinguished between cattle used for general purposes versus cattle used specifically for beef fattening (i.e. for meat production). We also included ES stemming from woody plants, which local people largely depend on for different purposes, namely firewood and honey (Shumi et al. 2019). Khat (*Catha edulis,* locally produced stimulant plant) and coffee (*Coffea arabica*), which is either grown naturally and collected in the forest (semi-forest coffee), or grown commercially in plantations (plantation coffee), are the main cash crops in the study area (Manlosa et al. 2019).

ES potential provision modeling

By dividing the total provision by the total population in each kebele, we calculated the per capita provision.

Biodiversity: We used the mean total woody plant species richness at the kebele level as a proxy for biodiversity. We fitted cross-validated generalized linear models for both forest and farmland, relating data on total and forest-specialist woody plant species richness to indicators of human disturbance and environmental conditions, and then projected for the baseline and the four scenarios (Duguma et al. 2023).

Cereal crops (maize, sorghum, teff); livestock (beef, cattle):

To model cereal crops and livestock, we used LM (least squares regression) models based on surveyed kebele data (official data and expert estimates for area of production and total annual production of each kebele; Brück et al. 2023a), as well as LULC data and additional geographical data for each kebele (elevation, distance to town, distance to road, woreda; Duguma et al. 2022).

Productivity was calculated as production divided by production area. Distance to road, pasture area, forest area, and total kebele area were log transformed, and all variables center-scaled to improve model estimation.

LM (least squares regression) models were selected for modelling, as these are easier to interpret for expert validation. These were used to relate both current productivity and area of production to the baseline predictor variables. Pearson correlation was used to determine variables that should not be

included in the same LM model, as this complicates the interpretation used for model reduction and expert validation. Biplot figures, with the outcome and the predictor variables, were assessed to identify potentially influential variables and need for quadratics. Plots were also used to determine the likely correct form of the model (e.g. linear, or log-log). Several models were fit for each outcome variable, and compared (using performance::compare_performance). Potential models were then pragmatically selected using principles of forwards and backwards selection, and based on hypothesized and identified influential variables, quadratics, and hypothesized interactions. Final models were selected based on their fit with data, check of model assumptions, and parsimony (fewer variables), as well as a visual check that the models produced sensible outcomes when projected to the current and future landscape scenarios.

Selected models were then projected for both the baseline and the scenarios. Total production was calculated as the estimated productivity times the estimated area of production. This methodology was selected to allow different influences on area and productivity per kebele in a pragmatic and easily interpretable form.

Tried and selected models (for productivity and area of production) for each ES:

The variables:

- Area_arable = area of arable land
- Area_forest = forest area
- Area_pasture = pasture area
- Distance_road = distance from the nearest road
- Distance_town = distance from the nearest town
- Elevm = mean elevation
- ES.py = the productivity of the respective ES
- ES_area = area under production of the respective ES
- Woreda = woreda (Gera, Gumay or Setema)

- Maize model
    - Productivity. Models tested (bold is selected model):
        - lm(maize.py ~ area_arable, data=DF)
        - **lm(maize.py ~ area_arable + poly(elevm,2), data=DF)**
        - lm(maize.py ~ poly(elevm,2) + area_arable + woreda, data=DF)
        - lm(maize.py ~ area_arable + poly(elevm,2) + distance_road + woreda, data=DF)
    - Area of production. Models tested (bold is selected model):
        - lm(maize_area ~ area_arable, data=DF)
        - lm(maize_area ~ elevm + area_arable, data=DF)
        - lm(maize_area ~ elevm*area_arable, data=DF)
        - **lm(maize_area ~ elevm + poly(area_arable,2), data=DF)**
        - lm(maize_area ~ elevm*poly(area_arable,2), data=DF)
- Sorghum model
    - Productivity. Models tested (bold is selected model):
        - lm(sorghum.py ~ area_arable, data=DF)
        - lm(sorghum.py ~ area_arable + elevm, data=DF)
        - lm(sorghum.py ~ elevm + area_arable + woreda, data=DF)
        - lm(sorghum.py ~ area_arable + elevm + distance_road + woreda, data=DF)
        - **lm(sorghum.py ~ elevm + woreda, data=DF)**
        - lm(sorghum.py ~ elevm*woreda, data=DF)
    - Area of production. Models tested (bold is selected model):
        - lm(sorghum_area ~ area_arable, data=DF)
        - lm(sorghum_area ~ poly(elevm,2) + area_arable, data=DF)
        - lm(sorghum_area ~ poly(elevm,2)*area_arable, data=DF)
        - lm(sorghum_area ~ poly(elevm,2) + area_arable + woreda, data=DF)
        - lm(sorghum_area ~ poly(elevm,2)*area_arable + woreda, data=DF)
        - lm(sorghum_area ~ poly(elevm,2) + area_arable + woreda + area_pasture, data=DF)
        - **lm(sorghum_area ~ poly(elevm,2) + poly(area_arable,2) + woreda, data=DF)**

- Teff model
  - Productivity. Models tested (bold is selected model):
    - lm(teff.py ~ elevm, data=DF)
    - lm(teff.py ~ elevm + distance_road, data=DF)
    - lm(teff.py ~ elevm + woreda, data=DF)
    - **lm(teff.py ~ elevm + woreda + area_arable, data=DF)**
  - Area of production. Models tested (bold is selected model):
    - lm(teff_area ~ area_arable, data=DF)
    - lm(teff_area ~ elevm + area_arable, data=DF)
    - lm(teff_area ~ elevm + poly(area_arable,2), data=DF)
    - lm(teff_area ~ elevm*area_arable, data=DF)
    - lm(teff_area ~ elevm*area_arable + woreda, data=DF)
    - lm(teff_area ~ elevm + poly(area_arable,2) + woreda, data=DF)
    - lm(teff_area ~ elevm + area_arable + area_forest + woreda + poly(distance_town,2), data=DF)
    - lm(teff_area ~ elevm + poly(area_arable,2) + woreda + poly(distance_town,2), data=DF)
    - **lm(teff_area ~ elevm + area_arable + woreda + poly(distance_town,2), data=DF)**
- Cattle model
  - Productivity. Models tested (bold is selected model):
    - lm(cattle.py ~ 1, data=DF)
    - lm(cattle.py ~ elevm, data=DF)
    - lm(cattle.py ~ elevm + distance_town, data=DF)
    - lm(cattle.py ~ elevm*distance_town, data=DF)
    - lm(cattle.py ~ distance_town, data=DF)
    - lm(log(cattle.py) ~ 1, data=DF)
    - **lm(log(cattle.py) ~ elevm, data=DF)**
    - lm(log(cattle.py) ~ elevm + distance_town, data=DF)
    - lm(log(cattle.py) ~ elevm*distance_town, data=DF)
    - lm(log(cattle.py) ~ distance_town, data=DF)
  - Area of production. Models tested (bold is selected model):
    - lm(log1p(cattle_area) ~ area_pasture + distance_town, data=DF)
    - lm(log1p(cattle_area) ~ poly(area_pasture,2) + distance_town, data=DF)
    - lm(log1p(cattle_area) ~ poly(area_pasture,2) + distance_road + distance_town, data=DF)
    - lm(log1p(cattle_area) ~ area_pasture * distance_town, data=DF)
    - lm(log1p(cattle_area) ~ area_pasture + distance_town + elevm, data=DF)
    - lm(log1p(cattle_area) ~ area_pasture * distance_town + elevm, data=DF)
    - lm(log1p(cattle_area) ~ area_pasture + distance_town + area_forest, data=DF)
    - lm(log1p(cattle_area) ~ area_pasture * distance_town + area_forest, data=DF)
    - **lm(log1p(cattle_area) ~ area_pasture * distance_town + area_forest + woreda, data=DF)**
- Beef model
  - Productivity. Models tested (bold is selected model):
    - **lm(beef.py ~ 1, data=DF)**
    - lm(beef.py ~ area_pasture, data=DF)
    - lm(beef.py ~ area_pasture + elevm, data=DF)
    - lm(beef.py ~ area_pasture + elevm + distance_town, data=DF)
    - lm(beef.py ~ (area_pasture + elevm + distance_town)^2, data=DF)
    - lm(beef.py ~ area_pasture*elevm*distance_town, data=DF)
  - Area of production. Models tested (bold is selected model):
    - lm(log1p(beef_area) ~ poly(area_pasture,2), data=DF)
    - lm(log1p(beef_area) ~ poly(area_pasture,2) + elevm, data=DF)
    - **lm(log1p(beef_area) ~ poly(area_pasture,2) + elevm + woreda, data=DF)**
    - lm(log1p(beef_area) ~ poly(area_pasture,2) + elevm + woreda + area_forest, data=DF)
    - lm(log1p(beef_area) ~ poly(area_pasture,2) * elevm + woreda, data=DF)
    - lm(log1p(beef_area) ~ area_pasture + elevm + area_forest + woreda, data=DF)
    - lm(log1p(beef_area) ~ area_pasture * elevm + area_forest + woreda, data=DF)
    - lm(log1p(beef_area) ~ poly(area_pasture,2) + elevm + area_forest + woreda, data=DF)
    - lm(log1p(beef_area) ~ poly(area_pasture,2) * elevm + area_forest + woreda, data=DF)

Firewood: We used the mean woody plant species abundance (that provide firewood) at the kebele level as a proxy. Negative binomial models for forest and farmland were based on LULC maps (derived from the narrative scenarios), household survey data on woody plant use, data on woody species distribution collected using ecological field surveys in different land-uses, as well as topographic

variables and human disturbance variables generated from LULC maps, and then projected for the baseline and the four scenarios (Duguma et al. Under Review).

Honey: We used the mean woody plant species abundance (that provide bee forage) at the kebele level as a proxy. For modeling, we followed the same approach as for firewood (Duguma et al. Under Review)

Khat: For each kebele, we multiplied khat area at the baseline and in the scenarios (from LULC maps; Duguma et al. 2022) by an average productivity of 0.51 t/ha (reported productivity in Oromia region in 2020/21 for peasant holdings; Central Statistical Agency of Ethiopia 2021).

Plantation coffee: For each kebele, we multiplied coffee plantation area at the baseline and in the scenarios (from LULC maps; Duguma et al. 2022) by an average productivity of 0.926 t/ha (reported productivity in Oromia region in 2020/21 for commercial farms; Central Statistical Agency of Ethiopia 2022).

Semi-forest coffee: We used a series of interlinked models, capturing key production chain nodes of coffee presence, management intensity, and expected yield, parameterized by data from three independent field studies (Shumi et al. 2019; Beche et al. 2022; Zewdie et al. 2022). We projected these for the baseline and the scenarios.

Spatial predictor variables included *elevation*, *slope*, heat load index (*hli*), topographic wetness index (*twi*), *forest type* (a binary variable of whether the forest existed 15 years prior or not), *distance* (from the forest edge), and percent woody vegetation in a 2km radius (*pwv2km*), and *woreda* (Duguma et al. 2022). All variables were examined and transformed if necessary to improve model performance.

To select candidate models, we focused on developing expert-driven, pragmatic (simple) models for each of the components, to be easily interpretable, and minimize the possibility of over-parameterization through the whole model.

<u>Coffee presence</u> was defined as a confirmed location of *Coffea arabica*, including both wild plants and those in managed forest areas, while absence was noted where the surveys did not record a presence. Coffee presence was modeled with a binomial GLM (using stats::glm):

```
glm(formula = coffee_presence ~ poly(elevation, 2) * hli, family = binomial, data = tdata)
```

<u>Coffee management intensity</u> was classified into 6 classes:

- A0: No coffee present, and therefore no management
- A1: Wild forest coffee with low/chance coffee abundance, no management and rarely harvested
- A2: Wild forest coffee with higher coffee abundance, no management and rarely harvested
- B: Wild forest coffee with minimal management (or historical but no current management), and more regular harvest
- C: Managed forest coffee with low-intensity management, including infilling (using local seedlings), pruning, slashing of competing understory, and minor reduction of overstory cover.
- D: Forest-plantation with relatively heavy management of the shade tree level, regular slashing and pruning, infilling (potentially with commercial cultivars), and typically ground-shrub layers aside from coffee removed completely

Coffee management was modeled using a cumulative link model (using ordinal::clm). The model to separate class D from other classes:

glm(formula = mgmtD ~ invPwv2km * pred_PA * forestType, family = binomial, data = tdata %>% filter(pred_PA > 0))

The model for management class:

ordinal::clm(formula = management_type2 ~ distance + pred_PA * W_NAME + pred_D, data = tdata %>% filter(pred_PA > 0))

Coffee yield was observed during fieldwork for all three included studies and was modelled using a zero inflated mixed model (using glmmTMB::glmmTMB):

glmmTMB(log1p(yield) ~ invPwv2km + pred_mgmt2_b * W_NAME + (1|datasource), ziformula = ~ pred_mgmt2_b * invPwv2km, data = tdata %>% filter(pred_PA>0))

We used deterministic model versions to predict the expected yield as:

EY[i] <- (probability of coffee presence > 0.5) * (1 – probability of yield absence) * (prediction of yield given presence)

The interlinked models were evaluated relative to the input data, and then projected to the baseline and the scenarios. To project the models, landscape rasters were aggregated to a cell size of 100m x 100m (1ha) using the mode for categorical and mean for continuous variables, extracted landscape variables from forest areas only, transformed these based on the transformation and polynomial models developed above, predicted outcomes via *nimble*, and converted back into raster format for further processing.

ES Specialization

We divided total potential provision of each ES in each kebele by total kebele area. We then log transformed the data to account for right skewed distributions, and normalized to [0, 1] through min-max scaling across the baseline and all scenarios for each ES. We then calculated the degree of ES specialization through Simpson's index (for infinite samples) based on the adjusted potential provision data across all ES in each kebele, at the baseline and under the four scenarios (gini.simpson.C in R package diverse, v0.1.5, Guevara et al. 2016; Brück et al. 2023a).

Value types

Through a survey with 164 local participants in two kebeles of each archetype, we assessed the mean importance ascribed to 11 ES within four value types (direct use, exchange, relational, intrinsic; Brück et al. 2023b). We did not collect data on plantation coffee, since it is currently not present in the landscape. Instead, based on the scenario description for the "Mining green gold" scenario (Jiren et al. 2020), we assumed high exchange values ascribed to it by local people, because they earn an income through employment in plantations (highest other mean exchange value is 5.89 for semi-forest coffee, so we assumed an exchange value of 6 for plantation coffee), whereas all other value types were assumed to be zero, because local people cannot directly use or relate to it. For each kebele, at the baseline and the scenarios, we multiplied the level of ES potential provision per capita in septiles (-3 to 3) by these ascribed values for each ES, and then summed across ES for each value type.

Results

Table S1. Kebeles of the study area and their social-ecological archetypes.

Kebele	Archetype
Awisa Bilo	Woody vegetation
Bara Enchni	Pasture-cropland
Berarigo	Woody vegetation
Bereguda	Woody vegetation
Berwerenigo	Pasture-cropland
Bore Dedo	Pasture-cropland
Bore Gogo	Khat-cropland
Boricho Deka	Woody vegetation
Chanido	Pasture-cropland
Chefeta Yera	Pasture-cropland
Chira Town	Accessible-wealthy
Demu Kufi	Pasture-cropland
Difo Mani	Woody vegetation
Done	Pasture-cropland
Dora Ongo	Khat-cropland
Doradocha	Khat-cropland
Duseta	Khat-cropland
Efyacgi	Accessible-wealthy
Gatira Town	Accessible-wealthy
Gatokure	Accessible-wealthy
Gebakoro	Khat-cropland
Gedagute	Khat-cropland
Gela	Pasture-cropland
Gemina Dacho	Woody vegetation
Genida Chala	Accessible-wealthy
Gere Ifalo	Woody vegetation
Gesecha	Pasture-cropland
Gido Bere	Woody vegetation
Gina Chola	Khat-cropland
Gurariso	Woody vegetation
Gure Dako	Khat-cropland
Guribodage	Woody vegetation
Kecha Aniderancha	Pasture-cropland
Kele	Woody vegetation
Kesebedado	Khat-cropland
Kola Kinibibit	Khat-cropland
Kola Suja	Pasture-cropland
Komibolicha	Khat-cropland
Kuba Toba	Khat-cropland
Kubo Silech	Pasture-cropland
Kuda Kefo	Accessible-wealthy
Kudakunacho	Woody vegetation
Lima Tad	Accessible-wealthy
Masano	Khat-cropland
Muje	Khat-cropland
Nego Agu	Khat-cropland
Oba Toli	Woody vegetation
Sata Gona	Pasture-cropland
Secha	Khat-cropland
Sed Loya	Accessible-wealthy
Sedu	Pasture-cropland
Seta	Khat-cropland
Setema Kecha	Accessible-wealthy
Sheni Chemere	Woody vegetation
Shoni Belira	Accessible-wealthy
Sika	Woody vegetation
Sogesecha	Pasture-cropland
Solako	Pasture-cropland
Susatela	Pasture-cropland
Tinibachale	Khat-cropland
Toba Town	Accessible-wealthy
Tuma Teso	Woody vegetation
Wala	Woody vegetation
Wanija Kerisa	Accessible-wealthy
Wegecha	Khat-cropland
Yasera Pera	Woody vegetation

Detailed description of the results (Figure 3)

For the study area on average (first column in each scenario block), potential provision per capita was extremely high for khat in the “Gain over grain” scenario, for plantation coffee under the “Mining green gold” scenario, and for maize and teff under the “Food first” scenario. It was extremely low for plantation coffee under all scenarios, except the “Mining green gold” scenario. Potential provision per capita showed the biggest increase from the baseline for plantation coffee under the “Mining green gold” scenario, closely followed by the increase in plantation coffee under the “Gain over grain” scenario, whereas the biggest decreases were observed for beef and cattle under the “gain over grain” scenario, for woody vegetation related ES and livestock under the “Mining green gold” scenario, and for firewood and livestock under the “Food first” scenario.

With regard to the kebele archetypes, potential provision per capita showed sometimes contrasting levels for certain ES under the same scenario, e.g. under the “Food first” scenario, honey was extremely low for the pasture-cropland archetype, whereas they were high and extremely high for the woody vegetation archetype. However, observed changes in the archetypes were never opposite, meaning that if an ES decreased under one scenario for the study area on average, we saw either no change or a decrease for each archetype, but never an increase (and vice versa).

In the woody vegetation kebele archetype, potential provision per capita was extremely high for woody vegetation related ES (semi-forest coffee, biodiversity, firewood, honey) at the baseline, and under all scenarios except “Mining green gold”; for khat under the “Gain over grain” scenario for all archetypes; for plantation coffee under the “Mining green gold” scenario for all archetypes except khat-cropland; under the “Food first” scenario, for biodiversity, honey and all cereal crops for the woody vegetation archetype, for maize and sorghum for the accessible-wealthy archetype, for teff for the pasture-cropland archetype. Potential provision per capita was extremely low for plantation coffee for all archetypes under the “Coffee and conservation” and “Food first” scenarios; for maize for the khat-cropland archetype under the “Gain over grain” scenario; for semi-forest coffee and honey for the pasture-cropland archetype under the “Food first” scenario. We observed the biggest increase from the baseline for plantation coffee under the “Mining green gold” scenario (closely followed by “Gain over grain”) for all archetypes, and the biggest decrease for the accessible-wealthy archetype for woody vegetation related ES (biodiversity, firewood, honey) under the “Mining green gold” scenario.

Variation between kebeles within the same archetype was generally low, but moderate variation was observed for khat and teff for some of the archetypes, and for the woody vegetation archetype for few ES, whereas semi-forest and plantation coffee partly showed high variation (Figure A1).

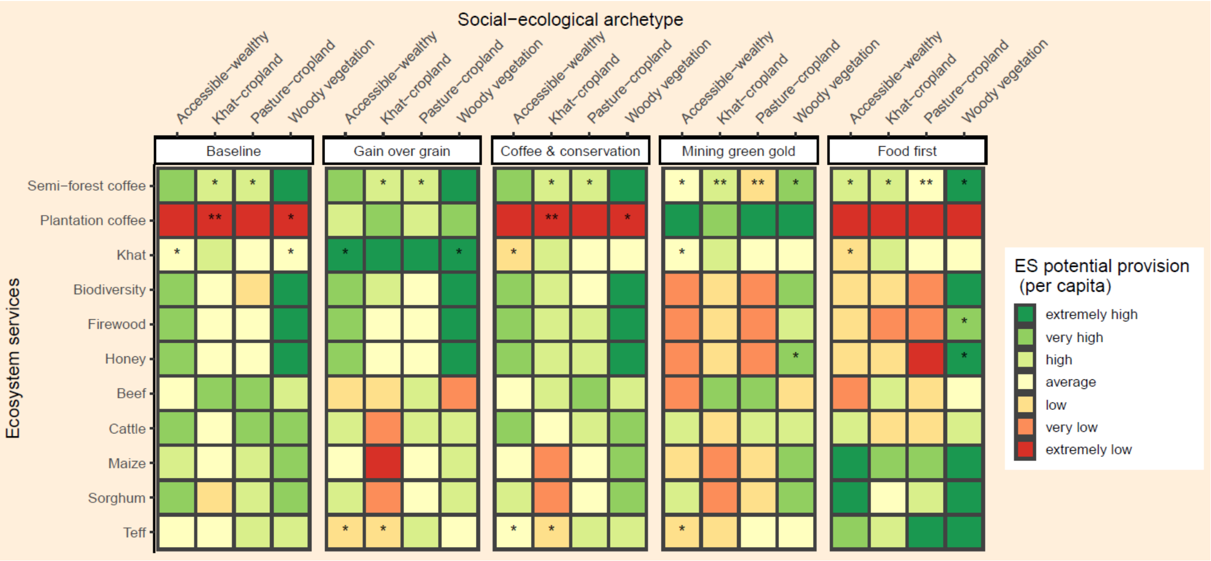

Figure S1. Relative levels of per capita potential provision of 11 locally important ES at the baseline and under four scenarios, and variation between kebeles within the same archetype. Variation was calculated through the coefficient of variation (standard deviation divided by mean) based on per capita provision of each ES across kebeles within each archetype. Coefficient of variation results across all ES and archetypes were then split into three equally sized intervals, indicating low, moderate (*) and high (**) variation.

Detailed description of the results (Figure 4)

For the study area on average (first column in each scenario block, Figure 4), intrinsic value was extremely low under the “Mining green gold” and the “Food first” scenario. ES specialization showed the biggest increase under the “Mining green gold” and the “Food first” scenarios, whereas the biggest decreases arose direct use and relational value under the “Mining green gold” scenario.

Under the same scenario, we sometimes observed contrasting levels of one aspect for different archetypes. Observed changes in the archetypes were never opposite, meaning that if an aspect decreased under one scenario for the study area on average, we saw either no change or a decrease for each archetype, but never an increase (and vice versa).

ES specialization was extremely high for the woody vegetation archetype under the “Mining green gold” scenario, and for the pasture-cropland archetype under the “Food first” scenario. Specialization was extremely low under the “Gain over grain” scenario for the accessible-wealthy archetype. For the woody vegetation archetype, direct use and relational value were extremely high at the baseline and under all scenarios (except “Mining green gold”), as well as exchange value under the “Gain over grain” scenario. Intrinsic value was extremely low for almost all archetypes under the two intensification scenarios. Specialization showed the biggest increase for the accessible-wealthy under the “Mining green gold” scenario and for the pasture-cropland archetype under the “Food first scenario”, and the biggest decrease was observed for the khat-cropland archetype under the “Gain over grain” scenario. The accessible-wealthy archetype showed the biggest decreases for direct use and relational value under the “Mining green gold” scenario.

The variation between kebeles within the same archetype was generally low, but moderate variation was sometimes observed, whereas intrinsic value partly showed high variation (Figure A2).

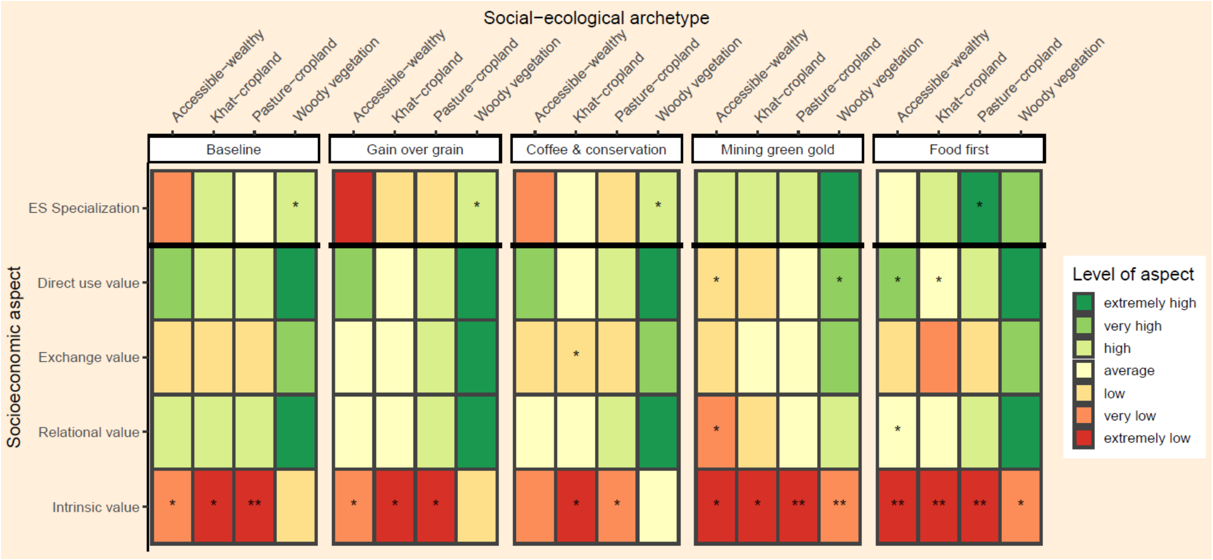

Figure S2. Relative levels of different socioeconomic aspects at the baseline and under four scenarios (ES specialization, direct use value, exchange value, relational value, intrinsic value), and variation between kebeles within the same archetype. Variation was calculated through the coefficient of variation (standard deviation divided by mean) based on specialization and value type data across kebeles within each archetype. Coefficient of variation results across all archetypes were then split into three equally sized intervals, indicating low, moderate (*) and high (**) variation.

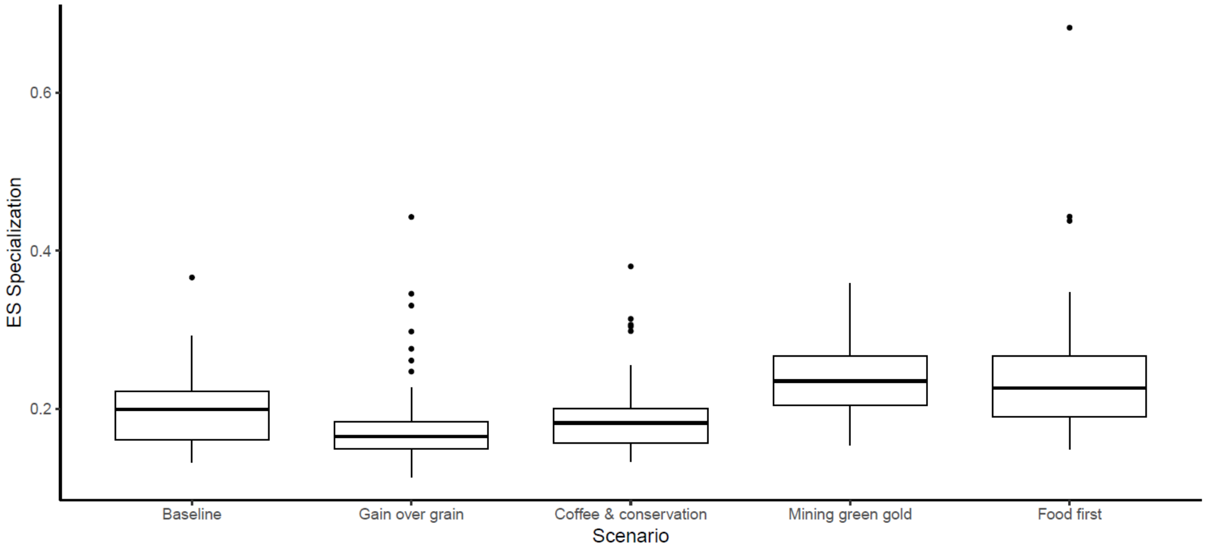

Figure S3. Boxplots of ES specialization at the baseline and for the four scenarios, across 66 kebeles.

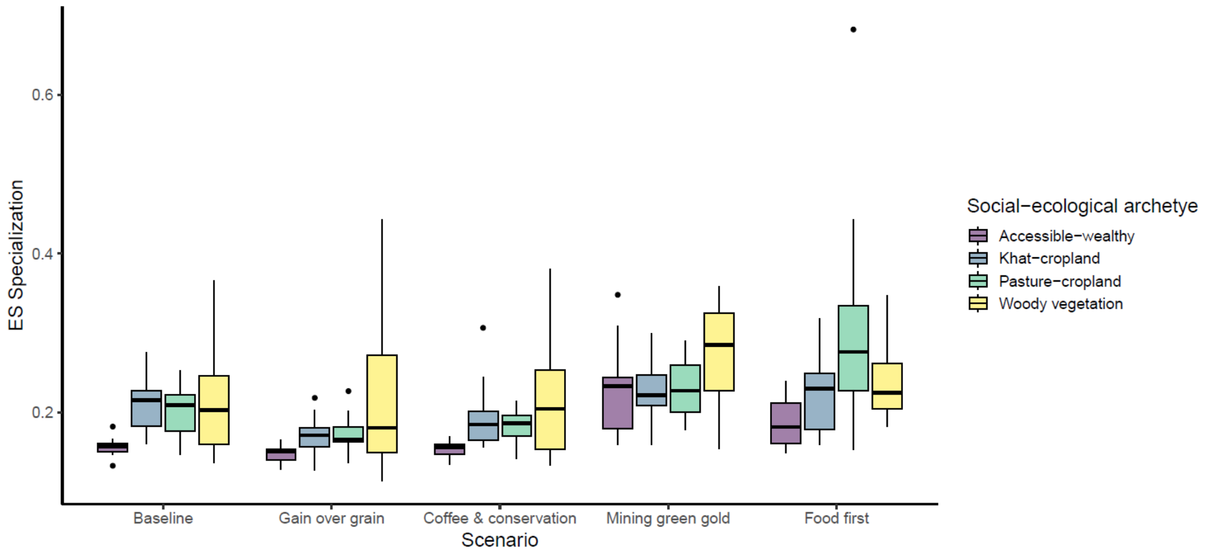

Figure S4. Boxplots of ES specialization at the baseline and for the four scenarios, across 66 kebeles, by social-ecological archetypes.

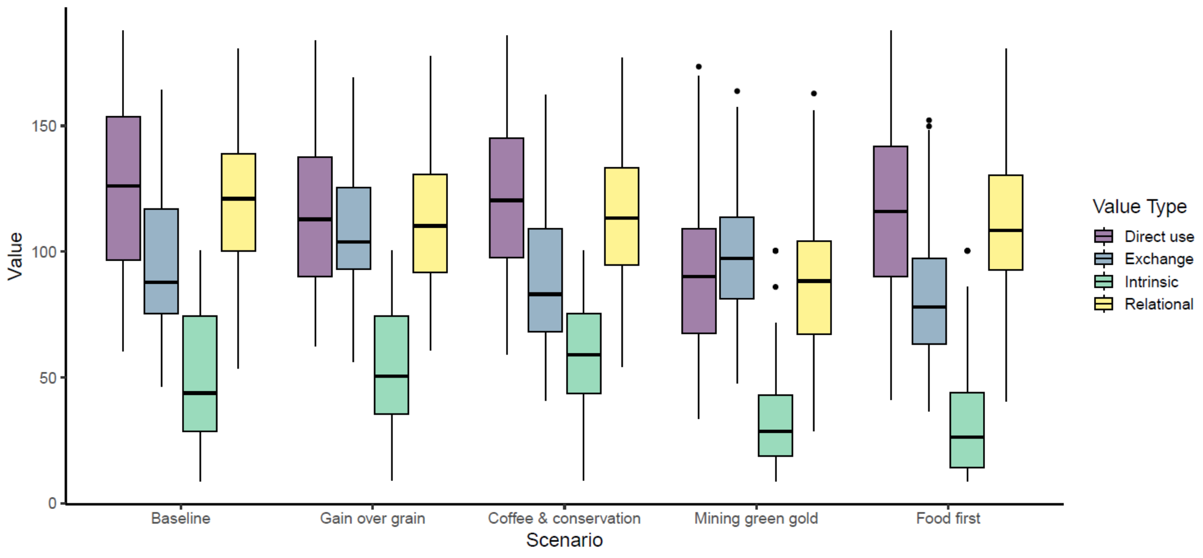

Figure S5. Boxplots of value importance at the baseline and for the four scenarios, across 66 kebeles, by value types.

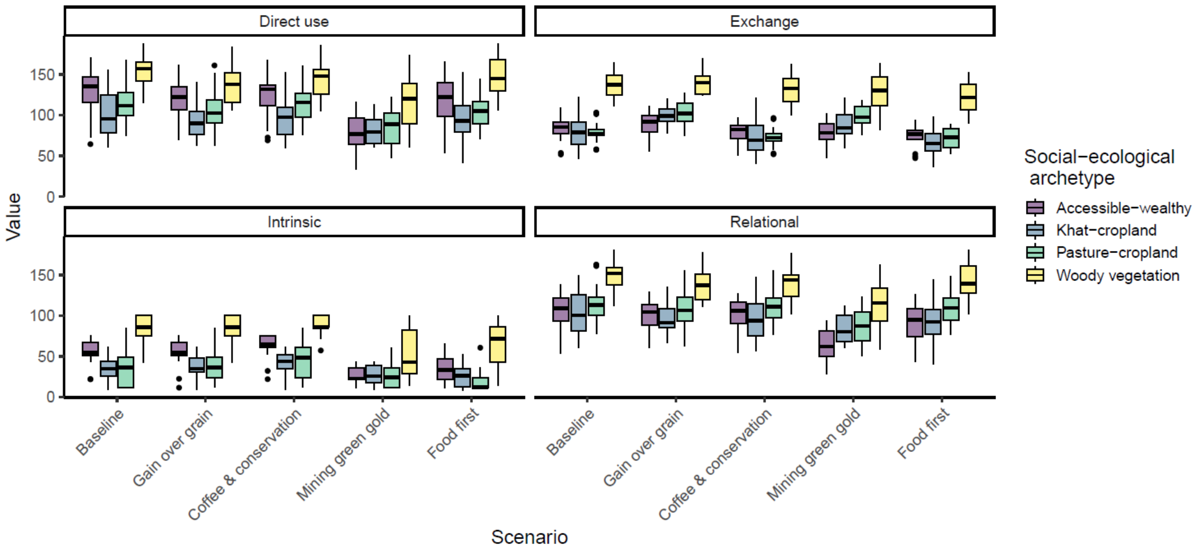

Figure S6. Boxplots of value importance at the baseline and for the four scenarios, across 66 kebeles, by value types and social-ecological archetypes.

References

Beche, D., A. Tack, S. Nemomissa, B. Warkineh, D. Lemessa, P. Rodrigues, J. Fischer, and K. Hylander. 2022. Spatial variation in human disturbances and their effects on forest structure and biodiversity across an Afromontane forest. *Landscape Ecology* 37: 493–510. doi: 10.1007/s10980-021-01395-4

Beenhouwer, M. de, L. Geeraert, J. Mertens, M. van Geel, R. Aerts, K. Vanderhaegen, and O. Honnay. 2016. Biodiversity and carbon storage co-benefits of coffee agroforestry across a gradient of increasing management intensity in the SW Ethiopian highlands. *Agriculture, Ecosystems and Environment* 222: 193–199. doi: 10.1016/j.agee.2016.02.017

Brooks, M.E., K. Kristensen, K.J. van Benthem, A. Magnusson, C.W. Berg, A. Nielsen, H.J. Skaug, M. Mächler, et al. 2017. glmmTMB Balances Speed and Flexibility Among Packages for Zero-inflated Generalized Linear Mixed Modeling. *The R Journal* 9: 378–400 (en).

Brück, M., J. Fischer, E. Law, J. Schultner, and D.J. Abson. 2023a. Drivers of ecosystem service specialization in a smallholder agricultural landscape of the Global South: a case study in Ethiopia. *Ecology and Society* 28. doi: 10.5751/ES-14185-280301

Brück, M., J. Schultner, B.B. Negash, F.D. Damu, and D.J. Abson. 2023b. Plural valuation in southwestern Ethiopia: Disaggregating values associated with ecosystems in a smallholder landscape. *People and Nature.* doi: 10.1002/pan3.10555

Central Statistical Agency of Ethiopia. 2021. Agricultural sample survey 2020/21: Volume I. Report on area and production of major crops (private peasant holdings, meher season).

Central Statistical Agency of Ethiopia. 2022. Statistical report on area and production of crops, and farm management practices: Large and medium scale commercial farms sample survey (2021/22).

Christensen, R. 2022. ordinal—Regression Models for Ordinal Data.

Duguma, D.W., E. Law, G. Shumi, J. Schultner, D.J. Abson, and J. Fischer. Under Review. Potential woody plant ecosystem services mapping in southwestern Ethiopia.

Duguma, D.W., J. Schultner, D.J. Abson, and J. Fischer. 2022. From stories to maps: translating participatory scenario narratives into spatially explicit information. *Ecology and Society* 27. doi: 10.5751/ES-13200-270213

Duguma, D.W., E. Law, G. Shumi, P. Rodrigues, F. Senbeta, J. Schultner, D.J. Abson, and J. Fischer. 2023. Spatial predictions for the distribution of woody plant species under different land-use scenarios in southwestern Ethiopia. *Landscape Ecology.* doi: 10.1007/s10980-023-01614-0

Guevara, M., D. Hartmann, and M. Mendoza. 2016. diverse: an R Package to Analyze Diversity in Complex Systems. *The R Journal* 8: 60–78.

Jiren, T.S., J. Hanspach, J. Schultner, J. Fischer, A. Bergsten, F. Senbeta, K. Hylander, and I. Dorresteijn. 2020. Reconciling food security and biodiversity conservation: participatory scenario planning in southwestern Ethiopia. *Ecology and Society* 25. doi: 10.5751/ES-11681-250324

Kassambara, A. 2020. ggpubr: 'ggplot2' Based Publication Ready Plots. R package version 0.4.0.

Lüdecke, D., M. Ben-Shachar, I. Patil, P. Waggoner, and D. Makowski. 2021. performance: An R Package for Assessment, Comparison and Testing of Statistical Models. *Journal of Open Source Software* 6: 3139. doi: 10.21105/joss.03139

Lüdecke, D. 2022. Data Visualization for Statistics in Social Science [R package sjPlot version 2.8.12]. Retrieved from https://CRAN.R-project.org/package=sjPlot.

Manlosa, A.O., J. Hanspach, J. Schultner, I. Dorresteijn, and J. Fischer. 2019. Livelihood strategies, capital assets, and food security in rural Southwest Ethiopia. *Food security* 11: 167–181 (eng). doi: 10.1007/s12571-018-00883-x

Pebesma, E. 2018. Simple Features for R: Standardized Support for Spatial Vector Data. *The R Journal* 10: 439. doi: 10.32614/RJ-2018-009
Peterson, R. 2021. Finding Optimal Normalizing Transformations via bestNormalize. *The R Journal* 13: 310. doi: 10.32614/RJ-2021-041
R Core Team. 2022. R: A language and environment for statistical computing. R version 4.2.1. Retrieved from https://www.R-project.org/.
Shumi, G., I. Dorresteijn, J. Schultner, K. Hylander, F. Senbeta, J. Hanspach, T.G. Ango, and J. Fischer. 2019. Woody plant use and management in relation to property rights: a social-ecological case study from southwestern Ethiopia. *Ecosystems and People* 15: 303–316. doi: 10.1080/26395916.2019.1674382
Wickham, H., M. Averick, J. Bryan, W. Chang, L. McGowan, R. François, G. Grolemund, A. Hayes, et al. 2019. Welcome to the Tidyverse. *Journal of Open Source Software* 4: 1686. doi: 10.21105/joss.01686
Wickham, H., and J. Bryan. 2023. Read Excel Files [R package readxl version 1.4.2]. Retrieved from https://CRAN.R-project.org/package=readxl.
Zewdie, B., A.J.M. Tack, B. Ayalew, M. Wondafrash, S. Nemomissa, and K. Hylander. 2022. Plant biodiversity declines with increasing coffee yield in Ethiopia's coffee agroforests. *Journal of Applied Ecology* 59: 1198–1208. doi: 10.1111/1365-2664.14130
